# Effect of Cooling Time on Demoulding Temperature, Shrinkage and Warpage of Injection-Moulded Polypropylene Parts

**DOI:** 10.3390/polym18151852

**Published:** 2026-07-29

**Authors:** Jan Kleinsorge, Yannick Luca Ollig-Gaul, Christian Hopmann

**Affiliations:** Institute for Plastics Processing (IKV) in Industry and Craft, RWTH Aachen University, Seffenter Weg 201, 52074 Aachen, Germany; jan.kleinsorge@ikv.rwth-aachen.de (J.K.);

**Keywords:** cooling time, injection moulding, polypropylene, post-consumer recycled material, process optimisation, warpage

## Abstract

In injection moulding, the cooling phase occupies 60–80% of the cycle time, making it the dominant factor for cost-effectiveness and a key driver of dimensional accuracy. Most previous work identified process windows that minimise warpage by simulation, indirectly inferring cooling time. This study experimentally quantifies the effect of cooling time on demoulding temperature and the resulting dimensional deviations. Housing parts were moulded from virgin and post-consumer recycled (PCR) polypropylene (PP). For PCR, a 3^3^ full-factorial design varied cooling time, cylinder temperature and mould temperature. Data were analysed by analysis of variance (ANOVA). For virgin PP, a nine-level design examined cooling time in finer detail. ANOVA revealed R^2^_CT_ of 0.54 for the effect of cooling time on demoulding temperature, identifying cooling time as the principal factor. For dimensional accuracy, the mould temperature showed the strongest effect (R^2^_MT_ = 0.44), while cooling-time changes from 0 s to 4 s showed a comparable effect (R^2^_CT_ = 0.36). For virgin PP, both the demoulding temperature and dimensional deviations decreased at a diminishing rate with increasing cooling time. Thus, a targeted adjustment of the cooling time in short time intervals leads to a significant improvement in dimensional accuracy without substantially compromising cost-effectiveness.

## 1. Introduction

Injection moulding is one of the most important manufacturing processes for processing polymers and is suitable for producing geometrically complex parts in large quantities. The cost effectiveness is largely determined by the cycle time. A considerable proportion of this time is taken up by the cooling phase [1], which typically accounts for 60% to 80% of the total cycle time [2,3,4]. Furthermore, the cooling phase has a direct influence on a moulded part’s quality. During the cooling phase, the plastic shrinks and can cause dimensional deviations. Non-uniform shrinkage leads not only to dimensional changes but also to changes in shape—warping [5]. If the cooling time is set too short, demoulding takes place before the material has solidified sufficiently. Without the confining effect of the mould, the material shrinks unhindered, which not only increases the degree of shrinkage but also the potential for warping.

Despite this central role, determining the optimal post-packing cooling time remains a challenge. The reason lies in the difficulty of characterising it. Formulas for estimating the required cooling time provide an initial approach to optimisation. Ballman and Shusman were among the first to derive a cooling time formula [6]. Subsequent work led to a multitude of further models, which are essentially based on the analytical solution of Fourier’s heat conduction equation for one-dimensional heat conduction and have been adapted to real process conditions using empirical correction factors. Stelson’s comparison of 26 references shows that many of these formulas are inconsistent [7]. Furthermore, all these approaches require knowledge of a demoulding temperature. Since the demoulding temperature is not an intrinsic material property, but depends on the specific process and part requirements, there is no standardised method for determining it. In practice, it is commonly estimated or approximated using thermo-mechanical parameters such as the Heat Deflection Temperature (HDT) or the Vicat temperature [7,8]. Both approaches inadequately represent the actual time-, geometry- and stress-dependent demoulding temperature, meaning that significant differences can arise between the calculated and the actually required cooling time.

For this reason, in practice, the parameters are predominantly determined empirically through injection moulding tests, based on recommended values [9]. Numerous studies focus on the influence of various process parameters on warpage using design of experiments (DoE), often employing the Taguchi method [10,11,12,13,14,15,16,17,18,19,20]. However, they rarely examine the influence of cooling time in detail and largely confine themselves to simulated warpage analyses rather than experimental measurements. The reported contributions of cooling time vary greatly depending on the material, geometry and parameter space under investigation.

Studies involving amorphous polymers tend to report lower sensitivity to variations in cooling time than those of semi-crystalline polymers [14,16,17]. Trinh et al. used simulation to investigate the influence of various process parameters on the warpage and shrinkage of an Acrylonitrile butadiene styrene (ABS) tensile specimen, employing a Taguchi parameter study and ANOVA analysis [14]. The results showed that melt temperature had the greatest influence on both warpage (R^2^_A_ = 0.67) and shrinkage (R^2^_A_ = 0.75). The variation in cooling time from 15 s to 25 s accounts for only R^2^_F_ = 0.03 of the warpage variation and a negligible proportion of the shrinkage variation.

Ren et al. investigated the influence of various process parameters on the shrinkage/warpage and tensile strength of overmoulded ABS/polycarbonate (PC) parts [12]. They do not specifically distinguish between shrinkage and warpage, but draw their conclusions from a combined value. Their experimental procedure also follows the Taguchi method and signal-to-noise (S/N) ratios. The variation in cooling time from 4 s to 12 s had the greatest influence on the shrinkage/warpage value and tensile strength. By selecting a cooling time of 12 s, the shrinkage/warpage value was reduced by 40%.

In contrast to amorphous polymers, studies reporting only a limited dependence on cooling time for semi-crystalline polymers are typically based on narrow cooling-time intervals or comparatively long cooling times [15,16,19]. Semi-crystalline polymers are therefore generally regarded as more sensitive to variations in cooling time. Kang carried out a Taguchi-based parametric simulation study on polypropylene and, using signal-to-noise (S/N) ratios and ANOVA, determined the optimal settings for injection time, holding pressure and time, and cooling time with regard to shrinkage and warpage [11]. The cooling time, which varied between 20 s and 40 s, showed the largest factor-specific contribution to simulated warpage with R^2^_CT_ = 0.45, followed by the holding pressure time with R^2^_HT_ = 0.30. Shrinkage, on the other hand, is influenced almost exclusively by the selected injection times, accounting for R^2^_IT_ = 0.97.

Sánchez et al. experimentally investigated the influence of cooling time, melt temperature and cooling conditions on the warpage of PP test specimens by using laser scanning [13]. The results showed that cooling time had the greatest influence. Increasing this from 12 s to 28 s reduced warpage by up to 70%. A higher melt temperature increased warpage, whilst asymmetric cooling had, surprisingly, little influence on warpage. The authors attributed this to a possibly poorly designed cooling layout.

Akçay and Şahin investigated the influence of cooling time and injection temperature on the warpage of an injection-moulded part made from a recycled polypropylene composite containing 43 wt% calcium carbonate [10]. The results show that cooling time has a significant influence on warpage and part weight. The authors attribute this to the fact that the cooling time influences the thermal history and temperature distribution within the mould, thereby playing a decisive role in the development of warpage. The lowest warpage values were achieved at an injection temperature of 225 °C and a cooling time of 32 s. However, no clear trend was discernible across the seven selected cooling time stages.

Chen et al. investigated the relationship between crystallisation behaviour, cooling time and moulded part shrinkage in PP [20]. By monitoring the melt temperature with an infrared sensor during solidification, the crystallisation time was determined and subsequently correlated with warpage measurements obtained at different cooling times. The results show a nearly linear decrease in warpage over the period between 5 s and 15 s, followed by an approximately constant level up to 30 s. The authors attributed this behaviour to the completion of crystallisation during processing and demonstrated that, once crystallisation was complete, no further significant changes in warpage occurred.

Previous studies show that cooling time has a significant influence on warpage. Nevertheless, most studies are limited to simulation-based investigations (Table 1) [11,14,16,17,18,19]. Without experimental validation, there is a risk that simulated distortions, whilst numerically consistent, may not reflect the actual processing conditions.

Furthermore, they typically consider only a small number of cooling time levels. Among the examined references, only Akçay and Şahin, and Chen et al., employ more than three levels for the cooling time [10,20]. In contrast, this study provides a detailed experimental investigation using finely graduated cooling time variations. In addition to quantifying the resulting warpage, the corresponding demoulding temperatures are measured experimentally, enabling a direct assessment of the relationship between cooling time, thermal state at demoulding, and dimensional accuracy. Linking those factors provides a more comprehensive understanding of the underlying mechanisms and supports a more precise optimisation of cycle time and part quality.

## 2. Materials and Methods

### 2.1. Materials

Polypropylene was selected because semi-crystalline plastics exhibit higher shrinkage and consequently greater potential for warping [5]. In addition, it represents the polymer with the highest global production share, accounting for approximately 17.8% of total plastic production [21]. One virgin polypropylene, PP 579S, from Saudi Basic Industries Corporation (SABIC), Riyadh, Saudi Arabia, and one PCR PP, Systalen PP-C44000 gr000, from Systec Plastics GmbH, Cologne, Germany, are used (Table 2). The selection of PCR is motivated by its increasing relevance due to industrial demands and regulatory requirements. Additionally, the comparison with virgin PP enables the investigation of the influence of material history on warpage behaviour.

### 2.2. Injection Moulding Setup and Data Acquisition System (DAQ)

**Figure 1:**
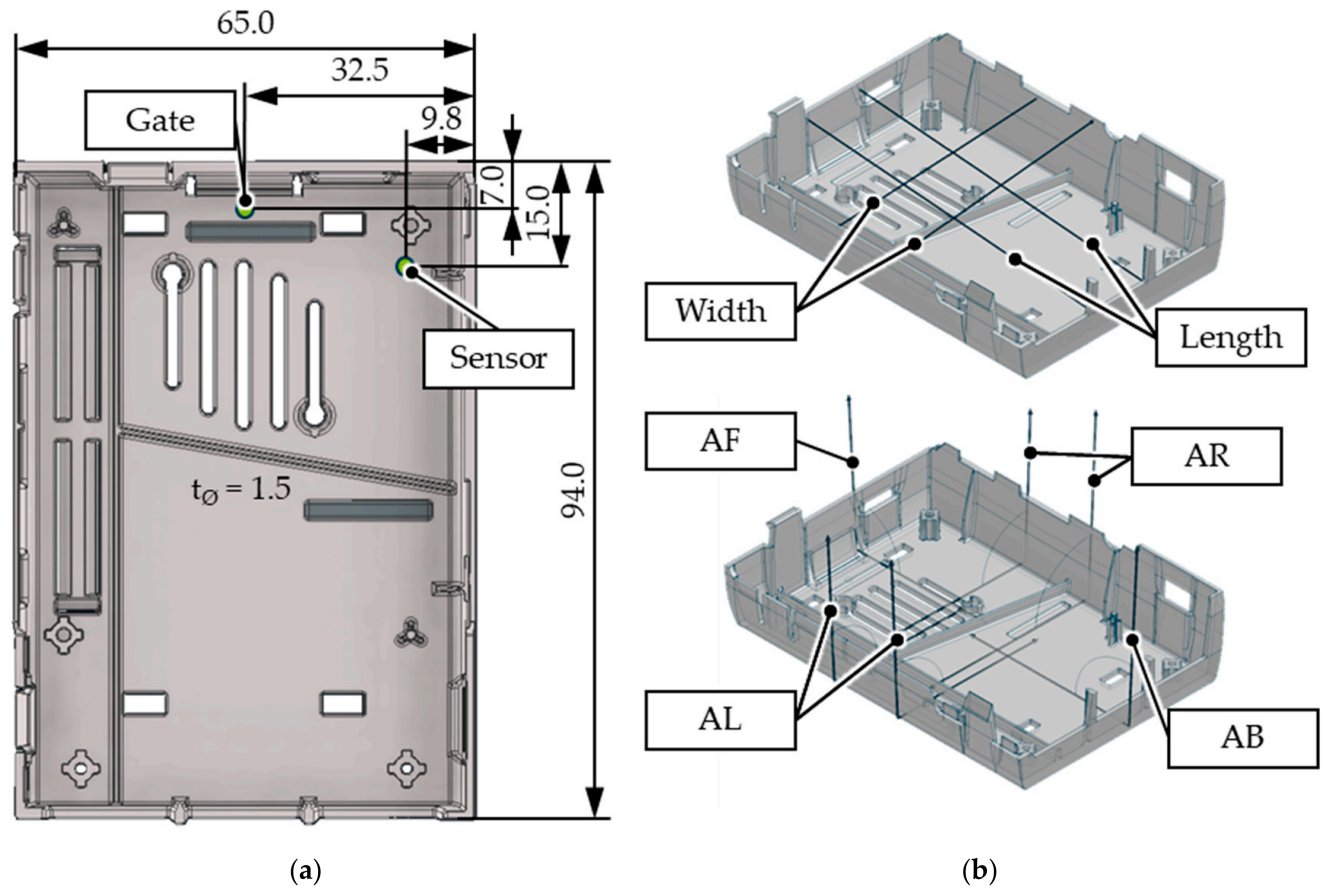
(**a**) Geometry and dimensions of the investigated housing, indicating the location of the gate and the temperature sensor. (**b**) Positions of the shrinkage and warpage measurements taken with the GOM ScanCobot: linear distances in the width and length directions, and angular measurements in the width direction—Angle Left (AL) and Angle Right (AR)—and in the length direction—Angle Front (AF) and Angle Back (AB).

All tests were carried out on the IntElect2 100/470-250 injection moulding machine manufactured by Sumitomo (SHI) Demag Plastics Machinery GmbH, Schwaig, Germany. The component produced is a housing manufactured using hot runner technology (Figure 1a).

A mould pressure and temperature sensor (Type K) with a diameter of 2.5 mm, model 6189A from the Kistler Group, Winterthur, Switzerland, is installed in the mould. The position of the sensor is shown in Figure 1a. The mould is temperature controlled using two Thermo-6 water temperature control units from HB-Therm AG, St. Gallen, Switzerland.

To obtain information about the solidification state during demoulding, an infrared thermometer, testo 835-H1, manufactured by Testo GmbH, Vienna, Austria, is used in addition to the sensor integrated into the mould. The infrared thermometer has a measurement range of −30 °C to +600 °C. Within the range of 0.0 °C to 99.9 °C, it measures with an accuracy of ±1 °C. Over the remaining measurement range, the accuracy is ±1.0% of the measured value. The device’s emissivity was kept at the default setting of 0.95, which is representative for most plastics. For each part, one manual measurement was taken at a reproducible distance from the part immediately after demoulding. The measurements were performed under reproducible environmental conditions to minimise the influence of ambient radiation.

### 2.3. Design of Experiments (DoE) in Injection Moulding

To quantitatively analyse the factors influencing shrinkage and warpage, a DoE was carried out in which several process parameters were systematically varied. The experimental design for the recycled material was chosen to compare the influence of various process parameters on warpage and to identify (demoulding) temperature fluctuations. For this purpose, three factors—which are post-packing cooling time, mould temperature and cylinder temperature—were selected, each with three levels (Table 3). The term “post-packing time”, as introduced here, refers to the set-parameter and thus only to the time following the holding phase. It should therefore be distinguished from the commonly used term “cooling time”, which begins as soon as the melt is injected and comes into contact with the mould.

The experimental design for virgin material aims to provide a detailed analysis of the cooling time effect. Therefore, only the mould temperature is varied across three levels and the post-packing cooling time across nine levels (Table 3). Furthermore, the holding pressure was adjusted for the two materials due to their different flow behaviours and resulting pressure transmission during the injection moulding process.

In order to be able to capture even short cooling times, the moulded part was demoulded immediately after the holding phase, and only then was fresh material dosed. Furthermore, the holding pressure time was deliberately set to a shorter duration. This results in a higher level of shrinkage across all test points. Based on the injection phase settings, an injection time of 0.4 s was achieved, resulting in a cooling time of 2.9 s before the remaining cooling period commenced. Ten parts were produced per test point.

### 2.4. Shrinkage and Warpage Measurement Setup

A GOM ScanCobot equipped with an optical 3D scanner, type ATOS Q, from Carl Zeiss GOM Metrology GmbH, Braunschweig, Germany, was used to measure the dimensional deviations of the moulded parts. In total, two distances each were measured across the width and length of the moulded part with reference to the nominal 3D geometry. Additionally, two angles between the base and each of the two long side walls (angle left (AL) and angle right (AR)), as well as one angle between the base and each of the short side walls (angle front (AF) and angle back (AB)), were measured. The positions of the characteristic values are shown in Figure 1b. The two measurements obtained for each dimension and angle category were subsequently averaged, yielding a single value for width, length, AL, AR, AF, and AB. For each of the test points, five of the ten parts were measured.

### 2.5. Statistical Analyses (ANOVA)

A full factorial three-way ANOVA was performed including all main effects and interaction terms using jamovi (version 2.7), the jamovi project, Australia. The normality of residuals and homogeneity of variances were assessed using Shapiro–Wilk and Levene’s tests. Significant deviations from these assumptions were detected for all tested metrics (*p* < 0.01), except for the demoulding temperature of recycled material (p_Levene_ = 0.17). Because the experimental design was fully balanced, with equal sample sizes across all factor combinations, the ANOVA was considered sufficiently robust to moderate assumption violations, particularly regarding Type-I errors [22,23]. However, the results should still be interpreted with caution. The significance level was set at α = 0.05. Due to the assumption violation, significance levels were confirmed using a permutation-based approach (5000 random permutations) with RjEditor, a jamovi module, yielding comparable significance patterns.

## 3. Results

The following section first examines the effect of cooling time on the interface and demoulding temperatures. It then describes the effect of cooling time on warpage.

### 3.1. Effect of Cooling Time on Interface and Demoulding Temperatures

**Figure 2:**
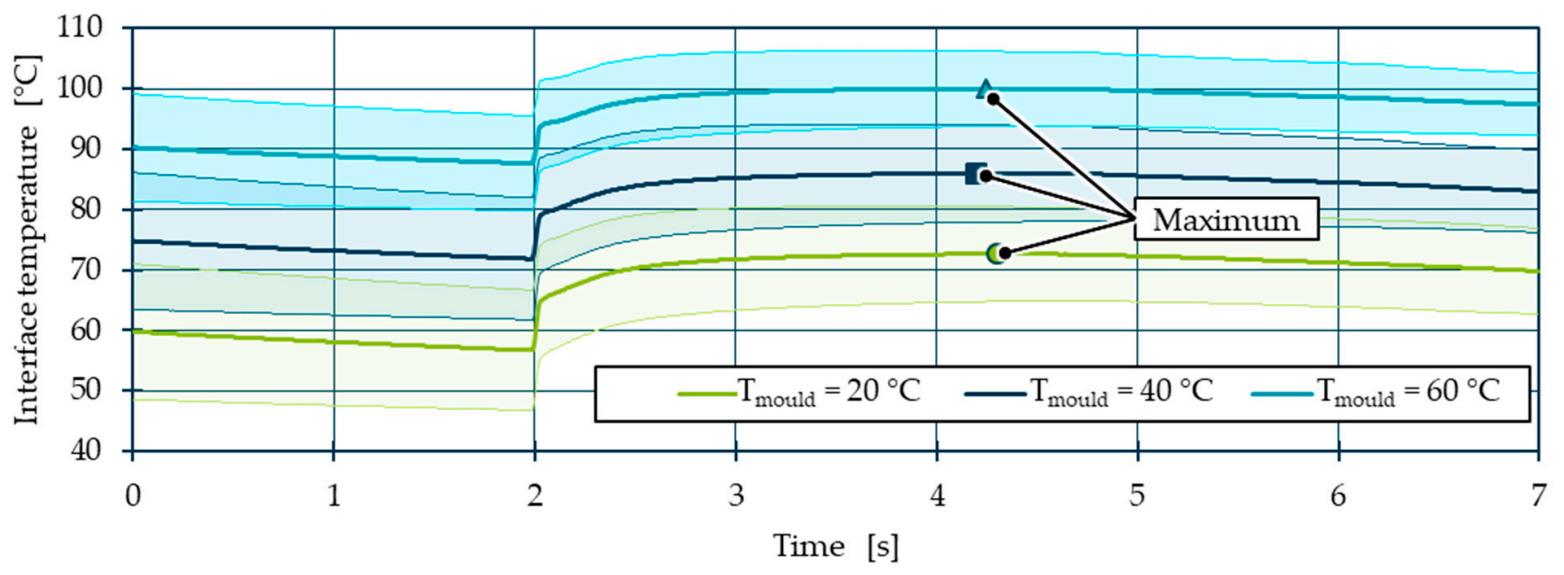
Average melt-to-mould interface temperature profile for the virgin polypropylene material at the three mould-temperature settings. The shaded bands around the mean curves indicate the standard deviation.

Figure 2 shows the interface temperature profile between the melt and the mould for the examined virgin material. The profile shown is an average of all test points for each of the three mould temperatures. The melt-to-mould interface temperature rises sharply as soon as the melt reaches the sensor until the marked maximum is reached. The temperature then falls until the next cycle. Although the height of the measured interface temperature curve changes with a change in mould temperature, the shape of the curve does not. An indication of this is the high Pearson correlation coefficient of r > 0.999 across all injection moulding cycles between a characteristic value within the curve, the maximum measured temperature, and the temperature integral. For this reason, the maximum measured temperature is used as an indicator of the curve.

**Figure 3:**
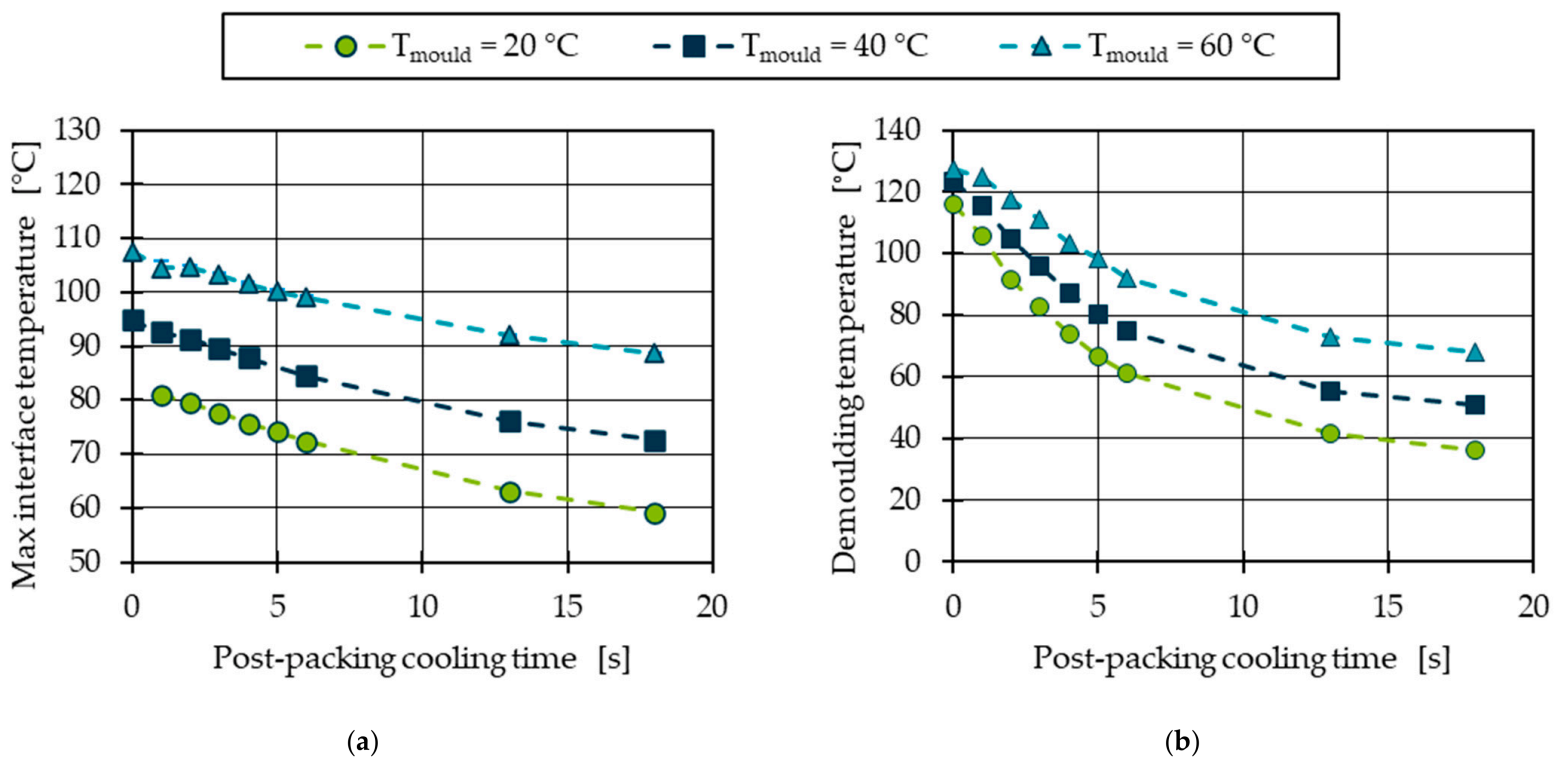
(**a**) Maximum melt-to-mould interface temperature and (**b**) demoulding temperature for the virgin polypropylene material at the three mould-temperature settings.

The maximum interface temperature decreases as the mould temperature falls (Figure 3a). Contrary to the expected decreasing trend, an almost linear curve can be observed over the remaining post-packing cooling time within the range under consideration. An asymptotic, decreasing behaviour may only become apparent at later stages. Due to errors in data recording, the measured values are missing for two test points. Averaged over the mould temperature, the maximum interface temperature decreases from 92.7 °C at a post-packing cooling time of 1 s to 73.5 °C at 18 s.

In contrast, the demoulding temperature shows a decreasing trend with a quadratic relationship between the post-packing cooling time and the demoulding temperature (Figure 3b). The demoulding temperature decreases from an average of 122.2 °C at a post-packing cooling time of 0 s to 51.8 °C at 18 s. Here too, the higher the mould temperature, the higher the demoulding temperature. As the measured values at 40 °C correspond almost exactly to the mean of the two outer stages, an approximately linear relationship between the mould temperature and the demoulding temperature is expected.

For the virgin material, the standard deviations of the maximum interface temperature—calculated separately for each experimental point—were pooled, resulting in a value of 0.31 °C, whilst that of the recycled material is 0.10 °C. The pooled standard deviation for the demoulding temperature of the virgin material is 1.00 °C; for the recycled material, the standard deviation is 0.76 °C. Although recycled materials are often associated with increased variability in processing behaviour, the present results indicate that this variability is not necessarily reflected in the investigated thermal process parameters [24]. Further material characterisation should be conducted, as the present analysis was limited to a single recyclate material.

The ANOVA carried out for the recycled material shows that variation in cooling time, with a coefficient of determination of R^2^_CT_ = 0.55, has the greatest influence on the demoulding temperature. Whilst the mould temperature, with R^2^_MT_ = 0.41, also explains a large proportion of the variation, the nozzle temperature, with R^2^_NT_ = 0.02, only contributes to a small proportion of the variation. For virgin material, the coefficient of determination for cooling time, at R^2^_CT_ = 0.80, is even more clearly the highest due to the large step size of the post-packing cooling time. The coefficient of determination for the mould temperature is R^2^_MT_ = 0.18 for virgin material.

### 3.2. Influence of the Cooling Time on Part Weight

**Figure 4:**
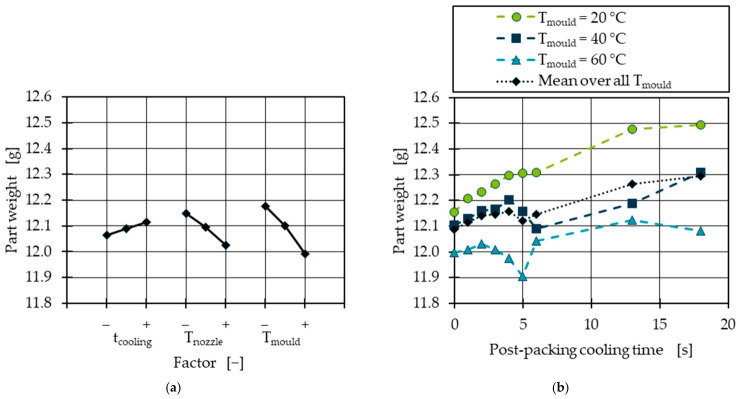
(**a**) Main effects plot showing the influence of post-packing cooling time (t_coling_), nozzle temperature (T_nozzle_) and mould temperature (T_mould_) on part weight made from recycled material. (**b**) Influence of post-packing cooling time and mould temperature on part weight for virgin material.

The weight of the recycled part increases from an average of 12.06 g at a post-packing cooling time of 0 s to 12.11 g at a post-packing cooling time of 4 s (Figure 4a). One possible cause is that the sealing point was not fully reached due to the short packing times. However, subsequent material transport from the screw pre-chamber during the post-packing cooling can largely be ruled out, as the experiments were carried out with a valve pin. Instead, a small material flow from the hot runner may have occurred. A similar observation was reported by Akçay and Şahin [10]. The nozzle and mould temperatures have the greatest influence on the weight. As the nozzle or mould temperature rises, the shot weight decreases. These observations are contrary to conventional expectations. One possible explanation is the increase in specific volume with rising temperature. As a higher specific volume corresponds to a lower density, this may result in a lower part weight. Similar findings were reported by Akçay and Şahin [10], as well as by Yang and Gao [25].

The part weight made from virgin material increases from an average of 12.09 g at a post-packing cooling time of 0 s to 12.30 g at 18 s (Figure 4b). The pooled standard deviation of each experimental point at a post-packing cooling time of 0 s is 0.012 g and 0.017 g at 18 s. For virgin material too, the weight decreases from 12.30 g at a mould temperature of 20 °C to 12.02 g as the mould temperature rises to 60 °C. This behaviour, which is comparable to that of the recycled material, can also be explained by a not fully reached sealing point and by the pvT behaviour. The irregularities observed in the weight curve at mould temperatures of 40 °C and 60 °C cannot yet be explained and require further investigation.

### 3.3. Influence of the Cooling Time on Dimensional Deviations (Warpage and Shrinkage)

The ANOVA results are shown in Table 4 exclusively for the experimental design using recycled material, as its expanded factor space allows for a better classification of the cooling time relative to other parameters. The values given in parentheses indicate coefficients of determination (R^2^) for effects that were not statistically significant. This criterion applies to both the *p*-values obtained from the ANOVA and those derived from the permutation-based approach. Changes in length and width reflect the combined effects of shrinkage and warpage, whereas the angular changes (AL, AR) provide a more direct measure of warpage.

**Figure 5:**
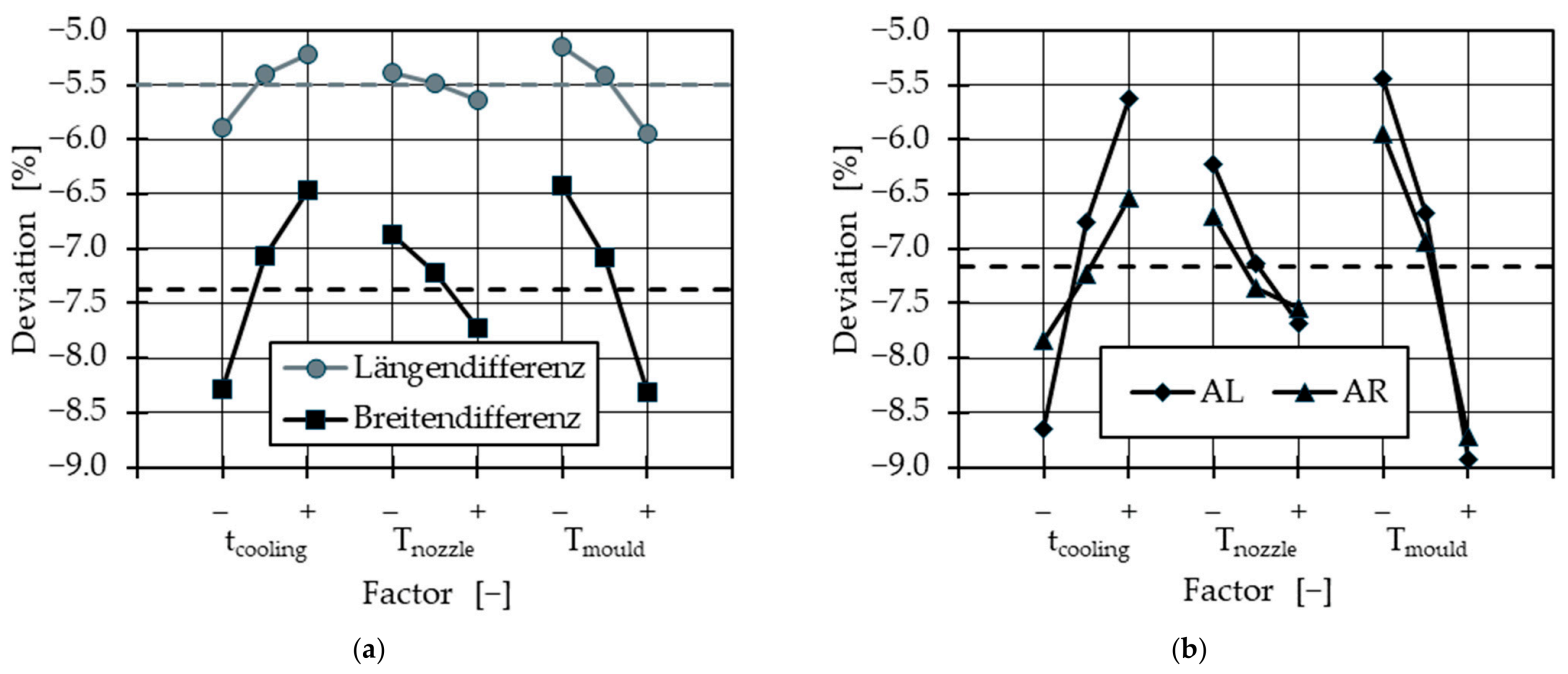
Main effects plot showing the influence of post-packing cooling time (t_coling_), nozzle temperature (T_nozzle_) and mould temperature (T_mould_) for the recycled material on (**a**) linear dimensions and on (**b**) width-related angular measurements. The dashed lines indicate the overall mean values.

The measured distances generally exhibit the highest proportion of explained variance, whereas the angular characteristics show a larger proportion of unexplained variance. This is particularly pronounced for the right (AR), front (AF), and back (AB) angles. Although the explained variance of the right angle is also relatively low, it still demonstrates a clear response to variations in the investigated process parameters (Figure 5b). By contrast, the front and back angles exhibit both a high level of unexplained variance and only negligible changes across the investigated parameter range. The same characteristics were observed for the virgin material. Consequently, the front and back angles are considered unsuitable for further interpretation and are therefore excluded from the subsequent analysis.

In general, the shrinkage and warpage metrics show the greatest dependence on mould temperature. The influence of cooling time is comparable, whilst the melt temperature has only a minor effect on dimensional deviations. This can be seen in the coefficient of determination as well as in the main-effects plot (Figure 5). It should also be noted that the influence of mould temperature is high due to the large step size, whilst the cooling time steps were chosen to be small in comparison to those reported in the literature. Furthermore, the observed increase in part weight with increasing cooling time may also contribute to improved dimensional accuracy, as it indicates a more complete compensation of volumetric shrinkage.

Whilst dimensional deviations decrease as cooling time increases, they increase as nozzle or mould temperature rises (Figure 5). These findings are consistent with the literature [12,13,14]. The housing exhibits an average shrinkage of 7.27% in the width direction. The corresponding angular deviation of the long side walls (AL, AR) amounts to −7.11% on average. In the length direction, the average shrinkage is 5.5%, while the angular deviation of the short side walls (AF, AB) from their nominal values reaches an average of −1.83%. The more pronounced shrinkage in the width direction can be attributed to the greater unsupported wall length and the resulting higher compliance of the part. The difference in the deviation between the width and length directions is considerably smaller than the corresponding difference in angular deviations. This indicates that the directional dependence of warpage is more pronounced than that of shrinkage.

**Figure 6:**
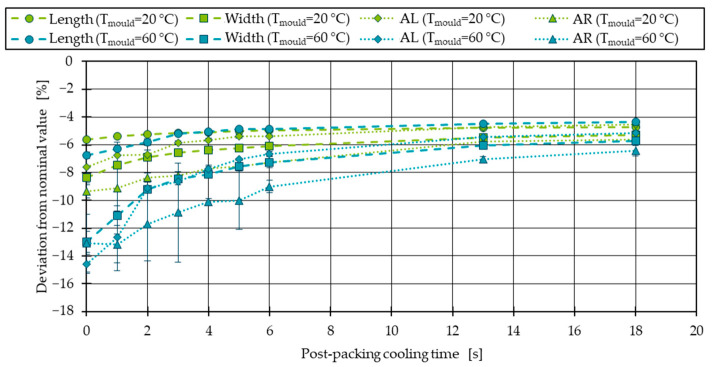
Deviations of shrinkage (linear dimensions) and warpage (angular measurements) as a function of post-packing cooling time for the investigated virgin polypropylene (PP).

For a more detailed analysis of the influence of the cooling time, the dimensional deviations of the virgin material are shown in Figure 6. Both shrinkage and warpage decrease on a declining basis as the post-packing cooling time increases. This finding is in agreement with the observation of Chen et al., who observed a strong reduction in warpage up to a cooling time of 15 s for one process condition, followed by a constant level of warpage [20]. According to Chen et al., this point indicates the completion of crystallisation. Similarly to the investigations on the recycled material, greater dimensional deviations are observed in the widthwise direction than in the lengthwise direction. Furthermore, as with the recycled material, greater dimensional deviations are evident at higher mould temperatures.

Higher mould temperatures lead to higher demoulding temperatures. The previously shown decreasing trend in the demoulding temperature over the cooling time shows a high correlation with the dimensional deviations. The Pearson coefficient is highest for length at 0.75 and lowest for the left angle at 0.61. This relationship is physically plausible, as a lower demoulding temperature indicates that a larger proportion of the total shrinkage has already occurred while the part remains constrained by the mould. Consequently, less post-mould shrinkage takes place after demoulding, reducing the potential for dimensional deviations and warpage. Furthermore, some of the improved dimensional accuracy at high cooling times can be attributed to the continued material flow. The Pearson correlation coefficient between the measured part weight and dimensional deviations is highest for the width at 0.64 and lowest for the length at 0.38.

**Figure 7:**
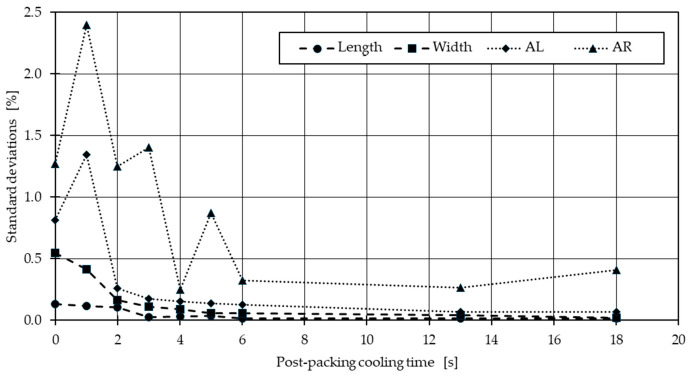
Pooled standard deviations of shrinkage (linear dimensions) and warpage (angular measurements) as a function of post-packing cooling time for the investigated virgin polypropylene (PP).

The standard deviations of the shrinkage and warpage values, pooled across the mould temperatures, are presented for the virgin material in Figure 7. The largest standard deviations were observed for AR, which also exhibited the greatest overall warpage. In contrast, the smallest standard deviations were found for the length, which generally showed the smallest deviations. For all values, the standard deviations decreased with increasing cooling time. This indicates that process- or material-related variations had a stronger influence on the warpage at shorter cooling times. A possible explanation is that the parts are demoulded at a higher temperature, before shrinkage has fully completed. As a result, shrinkage continues outside the constraint of the mould, making both shrinkage and the resulting warpage more susceptible to process- and material-related variations.

## 4. Conclusions and Outlook

The aim of this study was to examine the influence of cooling time on the dimensional accuracy of a housing component and its underlying thermal causes. Two material grades were investigated: a virgin PP and a recycled-grade PP. To assess the relative impact of cooling time compared with other process parameters, a full-factorial 3^3^ experimental design was performed on the recycled material, varying cooling time, nozzle temperature and mould temperature. In order to explore cooling times that fall below the usual recommendations, the packing time was intentionally reduced, which consequently resulted in greater shrinkage of the part.

For the recycled material, the most pronounced change in dimensional accuracy was observed when the mould temperature was increased from 20 °C to 60 °C. For the measured distances, the mould temperature effect accounted an average R^2^_MT_ of 0.44 corresponding to 44.41% of the total variance, whereas the modification of cooling time from 0 s to 4 s explained 36.57% of the variance. Dimensional deviations are generally larger in the widthwise direction than in the lengthwise direction because the walls there are longer and therefore more compliant. In addition, the difference in dimensional deviation between the widthwise and lengthwise directions is considerably smaller than the corresponding difference in angular deviation. This indicates that the directional dependence of warpage is more pronounced than that of shrinkage.

The effect of cooling time was investigated in greater detail on the virgin material using a nine-level experimental plan. Both the measured angles and the changes in linear distances exhibited a degressive decline as cooling time increased. This degressive behaviour of all dimensional-deviation indicators showed a strong correlation with the similarly degressive reduction in the demoulding temperature over cooling time, as reflected by Pearson coefficients ranging from 0.61 to 0.75, depending on the examined metric.

In summary, the close relationship between cooling time, demoulding temperature and dimensional deviations offers a wide range of avenues for further research into process optimisation or the development of adaptive process control strategies. In applications that rely on a short cycle time, even minor changes to the cooling time can lead to significant dimensional deviations. This is particularly relevant in view of the increasing use of recycled materials and the associated temperature fluctuations. By making a slight adjustment to the cooling time, temperature fluctuations at the time of demoulding could be compensated for, resulting in more uniform deformation.

## Figures and Tables

**Table 1:** A review of the literature on the influence of cooling time on warping.

Authors	Material Type	Cooling Time Levels	Warpage Data Source
Akçay and Şahin [10]	Semi-crystalline (PP composite)	7	Experimental results
Kang [11]	Semi-crystalline (PP)	3	Simulated results
Ren et al. [12]	Amorphous (ABS/PC overmoulding)	3	Experimental results
Sánchez et al. [13]	Semi-crystalline (PP)	3	Experimental results
Trinh et al. [14]	Amorphous (ABS)	3	Simulated results
Zhang et al. [15]	Amorphous (ABS)	3	Experimental results
Radwhan et al. [16]	Amorphous (ABS/PC)	3	Simulated results
Radwhan et al. [17]	Amorphous (ABS)	3	Simulated results
Trinh [18]	Semi-crystalline (PA6)	3	Simulated results
Lazim et al. [19]	Semi-crystalline (PP)	3	Simulated results
Chen et al. [20]	Semi-crystalline (PP)	8	Experimental results

**Table 2:** Material specifications.

	Virgin Material PP 579S	Recycled Material Systalen PP-C44000 gr000
MFR (230 °C/2.16 kg) (g/10 min)	47	12
Density (g/cm^3^)	0.905	0.92
Tensile strength (MPa)	35	25
Modulus of elasticity (MPa)	1900	1200

**Table 3:** Relevant processing parameters of the injection moulding process for both investigated materials.

	Virgin Material PP 579S	Recycled Material Systalen PP-C44000 gr000
Nozzle temperature (°C)	210	190, 210, 230
Mould temperature (°C)	20, 40, 60	20, 40, 60
Volumetric injection rate (cm^3^/s)	50	50
Holding pressure (bar)	320	400
Packing time (s)	2.5	2.5
Post-packing cooling time (s)	0, 1, 2, 3, 4, 5, 6, 13, 18	0, 2, 4
Screw peripheral speed (mm/s)	650	650

**Table 4:** Coefficient of determination (R^2^) for shrinkage and warpage of the recycled material.

	R^2^_cooling time_ (−)	R^2^_nozzle temperature_ (−)	R^2^_mould temperature_ (−)	Explained Variance (−)
Length	0.34	0.04	0.47	0.91
Width	0.39	0.08	0.42	0.97
Angle Left (AL)	0.28	0.06	0.37	0.84
Angle Right (AR)	(0.03)	(0.01)	0.15	0.36
Angle Front (AF)	0.05	(0.02)	0.06	0.37
Angle Back (AB)	(0.03)	(0.02)	(>0.01)	0.25

## Data Availability

The original contributions presented in this study are included in the article. Further inquiries can be directed to the corresponding author.

## Abbreviations

The following abbreviations are used in this manuscript:

AB	Angle back
ABS	Acrylonitrile butadiene styrene
AF	Angle front
AL	Angle left
AR	Angle right
ANOVA	Analysis of variance
DAQ	Data Acquisition System
DoE	Design of Experiments
HDT	Heat Deflection Temperature
MFR	Melt flow rate
PC	Polycarbonate
PCR	Post-consumer recycled
PP	Polypropylene
R^2^	Coefficient of determination
R^2^_CT_	Factor-specific coefficient of determination for the cooling time
R^2^_HT_	Factor-specific coefficient of determination for the holding pressure time
R^2^_IT_	Factor-specific coefficient of determination for the injection time
R^2^_MT_	Factor-specific coefficient of determination for the mould temperature
R^2^_NT_	Factor-specific coefficient of determination for the nozzle temperature
SABIC	Saudi Basic Industries Corporation
S/N	Signal-to-noise
t_cooling_	Post-packing cooling time
T_mould_	Mould temperature
T_nozzle_	Nozzle temperature
